# Dimethyl 2-[23-oxo-22,24-diphenyl-8,11,14-trioxa-25-aza­tetra­cyclo­[19.3.1.0^2,7^.0^15,20^]penta­cosa-2,4,6,15(20),16,18-hexaen-25-yl]but-2-enedioate

**DOI:** 10.1107/S1600536812015206

**Published:** 2012-04-13

**Authors:** Le Tuan Anh, Truong Hong Hieu, Anatoly T. Soldatenkov, Svetlana A. Soldatova, Victor N. Khrustalev

**Affiliations:** a Department of Chemistry, Vietnam National University, 144 Xuan Thuy, Cau Giay, Hanoi, Vietnam; b Organic Chemistry Department, Russian Peoples Friendship University, Miklukho-Maklaya St 6, Moscow 117198, Russian Federation; c X-Ray Structural Centre, A.N. Nesmeyanov Institute of Organoelement Compounds, Russian Academy of Sciences, 28 Vavilov St, B-334, Moscow 119991, Russian Federation

## Abstract

The title compound, C_39_H_37_NO_8_, is a product of the Michael addition of the cyclic secondary amine subunit of aza-14-crown-4 ether to dimethyl acetyl­enedicarboxyl­ate. The piperidinone ring exhibits a distorted chair conformation and the dimethyl acetyl­enedicarboxyl­ate fragment has a *cis* configuration with a dihedral angle of 56.61 (5)° between the two carboxyl­ate groups. The crystal packing is stabilized by the weak C—H⋯O hydrogen bonds.

## Related literature

For general background to the design, synthesis, chemical properties and applications of macrocyclic ligands in coordination chemistry, see: Hiraoka (1978 ▶); Pedersen (1988 ▶); Schwan & Warkentin (1988 ▶); Gokel & Murillo (1996 ▶); Bradshaw & Izatt (1997 ▶). For related compounds, see: Levov *et al.* (2006 ▶, 2008 ▶); Anh *et al.* (2008 ▶); Hieu *et al.* (2011 ▶); Khieu *et al.* (2011 ▶).
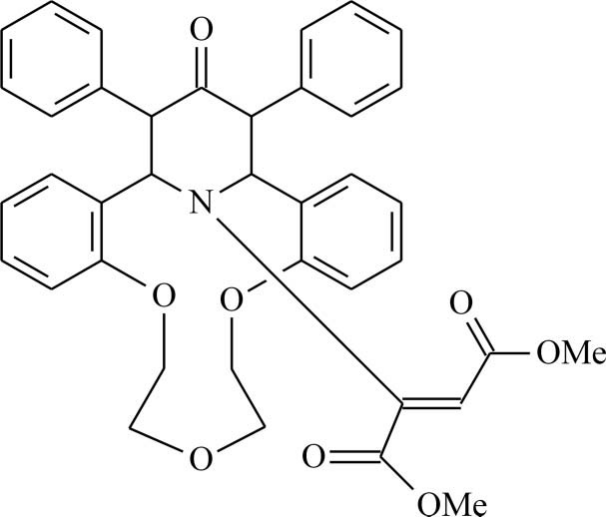


## Experimental

### 

#### Crystal data

C_39_H_37_NO_8_
*M* *_r_* = 647.70
Triclinic, 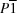

*a* = 10.9914 (6) Å
*b* = 11.7868 (6) Å
*c* = 13.7725 (7) Å
α = 114.306 (1)°
β = 91.211 (1)°
γ = 91.984 (1)°
*V* = 1623.91 (15) Å^3^
*Z* = 2
Mo *K*α radiation
μ = 0.09 mm^−1^
*T* = 100 K
0.28 × 0.22 × 0.20 mm

#### Data collection

Bruker APEXII CCD diffractometer
Absorption correction: multi-scan (*SADABS*; Sheldrick, 2003 ▶) *T*_min_ = 0.975, *T*_max_ = 0.982
19532 measured reflections
8601 independent reflections
7062 reflections with *I* > 2σ(*I*)
*R*_int_ = 0.028

#### Refinement

*R*[*F* ^2^ > 2σ(*F* ^2^)] = 0.042
*wR*(*F* ^2^) = 0.108
*S* = 1.00
8601 reflections
435 parameters
H-atom parameters constrained
Δρ_max_ = 0.38 e Å^−3^
Δρ_min_ = −0.29 e Å^−3^

### 

Data collection: *APEX2* (Bruker, 2005 ▶); cell refinement: *SAINT-Plus* (Bruker, 2001 ▶); data reduction: *SAINT-Plus*; program(s) used to solve structure: *SHELXTL* (Sheldrick, 2008 ▶); program(s) used to refine structure: *SHELXTL*; molecular graphics: *SHELXTL*; software used to prepare material for publication: *SHELXTL*.

## Supplementary Material

Crystal structure: contains datablock(s) global, I. DOI: 10.1107/S1600536812015206/cv5280sup1.cif

Structure factors: contains datablock(s) I. DOI: 10.1107/S1600536812015206/cv5280Isup2.hkl

Supplementary material file. DOI: 10.1107/S1600536812015206/cv5280Isup3.cml

Additional supplementary materials:  crystallographic information; 3D view; checkCIF report

## Figures and Tables

**Table 1:** Hydrogen-bond geometry (Å, °)

*D*—H⋯*A*	*D*—H	H⋯*A*	*D*⋯*A*	*D*—H⋯*A*
C6—H6⋯O3^i^	0.95	2.58	3.3982 (16)	145
C10—H10*A*⋯O1^ii^	0.99	2.44	3.2433 (16)	138
C12—H12*A*⋯O2^iii^	0.99	2.58	3.5345 (18)	162
C17—H17⋯O5^iv^	0.95	2.53	3.4409 (18)	160
C30—H30⋯O4^v^	0.95	2.53	3.2834 (16)	136
C41—H41*A*⋯O4^vi^	0.98	2.52	3.3758 (19)	145

Symmetry codes: (i) 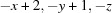; (ii) 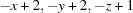; (iii) 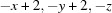; (iv) 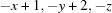; (v) 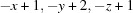; (vi) 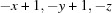.

**Fig. 1.**
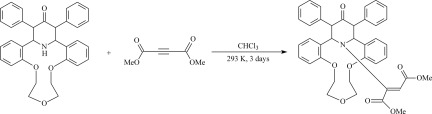
Michael addition of bis(benzo)-(β,β'-diphenyl-γ-piperidono)aza-14-crown-4 ether to dimethyl acetylenedicarboxylate.

**Fig. 2.**
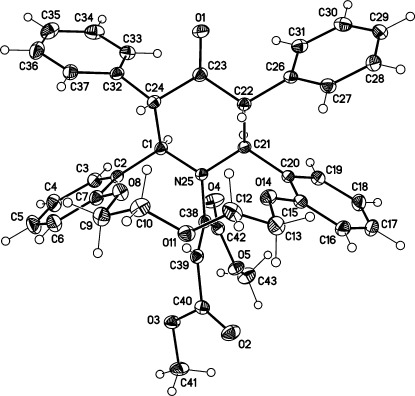
Molecular structure of I. Displacement ellipsoids are shown at the 50% probability level. H atoms are presented as small spheres of arbitrary radius.

## supplementary crystallographic information

### Comment

Design, synthesis and applications of macrocyclic ligands for coordination and supramolecular chemistry draw very great attention of investigators during the last forty years (Hiraoka, 1978; Pedersen, 1988; Gokel & Murillo, 1996; Bradshaw & Izatt, 1997). Recently we have developed the effective methods of synthesis of azacrown ethers containing piperidine (Levov *et al.*, 2006, 2008; Anh *et al.*, 2008), perhydropyrimidine (Hieu *et al.*, 2011) and perhydrotriazine subunits (Khieu *et al.*, 2011).

In attempts to apply this chemistry for obtaining of a macrocyclic ligand bringing the desirable functional groups, we studied the Michael addition of the cyclic secondary amine subunit of the crown ether to dimethyl acetylenedicarboxylate. The expected reaction is well known (Schwan & Warkentin, 1988), but might be highly hindered due to the steric reasons. We have found, however, that the expected *N*-vynilation proceeded smoothly with the formation of an *N*-maleinate derivative of the azacrown system.

The title compound, I, is a product of the Michael addition of the cyclic secondary amine subunit of the aza-14-crown-4 ether to dimethyl acetylenedicarboxylate (Figure 1). The title macromolecule includes the aza-14-crown-4-ether skeletal moiety and adopts a bowl conformation (Figure 2). The configuration of the C7—O8—C9—C10—O11—C12—C13—O14—C15 polyether chain is t–g^(-)^–t–t–g^(+)^–t (t = *trans*, 180°; g = *gauche*, ±60°). The piperidinone ring of the bicyclic fragment have a slightly flattenned *chair* conformation. The dihedral angle between the planes of the benzene rings fused to the aza-14-crown-4-ether moiety is 57.14 (4)°. The phenyl rings at the C22 and C24 carbon atoms occupy the sterically favorable equatorial positions and are rotated to each other by 34.06 (6)°. The carboxylate substituents are rotated to each other by 56.61 (5)°. The volume of the internal cavity of macrocycle I is approximately equal to 66 Å^3^.

The molecule of I possesses four asymmetric centers at the C1, C21, C22 and C24 carbon atoms and can have potentially numerous diastereomers. The crystal of I is racemic and consists of enantiomeric pairs with the following relative configuration of the centers: *rac*-1*R**,21*S**,22*R**,24*S**.

In the crystal, the molecules of I are bound to each other by weak C—H···O hydrogen bonding interactions (Table 1) into three-dimensional framework.

### Experimental

Dimethyl acetylenedicarboxylate (0.14 g, 0.99 mmol) was added to a solution of bis(benzo)-(β,β'-diphenyl-γ-piperidono)aza-14-crown-4 ether (0.5 g, 0.99 mmol) in chloroform (20 ml). The reaction mixture was stirred at 293 K for 3 days (monitoring by TLC until disappearance of the starting organic compounds spots). At the end of the reaction, the formed precipitate was separated, washed with cold chloroform (50 ml) and re-crystallized from ethanol to give 0.61 g of colourless crystals of I. Yield is 94%. *M*.p. = 514–516 K. IR (KBr), ν/cm^-1^: 1600, 1632, 1713. ^1^H NMR (CDCl_3_ , 400 MHz, 300 K): δ = 3.34 and 3.41 (both s, 3H each, CH_3_), 4.08,4.21 and 4.27 (all m, 4H, 2H and 2H, respectively, OCH_2_CH_2_O), 4.73 (d, 2H, H22 and H24, *J* = 10.8), 5.19 (d, 2H, H1 and H21, *J* = 10.8), 6.52 and 6.64 (both m, 2H and 4H, respectively, H_arom_), 6.97 (c, 1H, O_2_C–CH=C–CO_2_), 6.99–7.14 (m, 12H, H_arom_). Anal. Calcd for C_39_H_37_NO_8_: C, 72.32; H, 5.76; N, 2.16. Found: C, 72.28; H, 5.87; N, 2.12.

### Refinement

The hydrogen atoms were placed in calculated positions with C—H = 0.95–1.00 Å and refined in the riding model with fixed isotropic displacement parameters [*U*_iso_(H) = 1.5*U*_eq_(C) for the methyl groups and 1.2*U*_eq_(C) for the other groups].

### Crystal data

**Table:**

C_39_H_37_NO_8_	*Z* = 2
*M**_r_* = 647.70	*F*(000) = 684
Triclinic, *P*1	*D*_x_ = 1.325 Mg m^−^^3^
Hall symbol: -P 1	Mo *K*α radiation, λ = 0.71073 Å
*a* = 10.9914 (6) Å	Cell parameters from 8458 reflections
*b* = 11.7868 (6) Å	θ = 2.4–32.6°
*c* = 13.7725 (7) Å	µ = 0.09 mm^−^^1^
α = 114.306 (1)°	*T* = 100 K
β = 91.211 (1)°	Prism, colourless
γ = 91.984 (1)°	0.28 × 0.22 × 0.20 mm
*V* = 1623.91 (15) Å^3^	

### Data collection

**Table:**

Bruker APEXII CCD diffractometer	8601 independent reflections
Radiation source: fine-focus sealed tube	7062 reflections with *I* > 2σ(*I*)
Graphite monochromator	*R*_int_ = 0.028
φ and ω scans	θ_max_ = 29.0°, θ_min_ = 1.6°
Absorption correction: multi-scan (*SADABS*; Sheldrick, 2003)	*h* = −14→14
*T*_min_ = 0.975, *T*_max_ = 0.982	*k* = −16→16
19532 measured reflections	*l* = −18→18

### Refinement

**Table:**

Refinement on *F*^2^	Primary atom site location: structure-invariant direct methods
Least-squares matrix: full	Secondary atom site location: difference Fourier map
*R*[*F*^2^ > 2σ(*F*^2^)] = 0.042	Hydrogen site location: inferred from neighbouring sites
*wR*(*F*^2^) = 0.108	H-atom parameters constrained
*S* = 1.00	*w* = 1/[σ^2^(*F*_o_^2^) + (0.049*P*)^2^ + 0.6*P*] where *P* = (*F*_o_^2^ + 2*F*_c_^2^)/3
8601 reflections	(Δ/σ)_max_ = 0.001
435 parameters	Δρ_max_ = 0.38 e Å^−^^3^
0 restraints	Δρ_min_ = −0.29 e Å^−^^3^

### Special details

**Table:**

Geometry. All e.s.d.'s (except the e.s.d. in the dihedral angle between two l.s. planes) are estimated using the full covariance matrix. The cell e.s.d.'s are taken into account individually in the estimation of e.s.d.'s in distances, angles and torsion angles; correlations between e.s.d.'s in cell parameters are only used when they are defined by crystal symmetry. An approximate (isotropic) treatment of cell e.s.d.'s is used for estimating e.s.d.'s involving l.s. planes.
Refinement. Refinement of *F*^2^ against ALL reflections. The weighted *R*-factor *wR* and goodness of fit *S* are based on *F*^2^, conventional *R*-factors *R* are based on *F*, with *F* set to zero for negative *F*^2^. The threshold expression of *F*^2^ > σ(*F*^2^) is used only for calculating *R*-factors(gt) *etc*. and is not relevant to the choice of reflections for refinement. *R*-factors based on *F*^2^ are statistically about twice as large as those based on *F*, and *R*- factors based on ALL data will be even larger.

### Fractional atomic coordinates and isotropic or equivalent isotropic displacement parameters (Å^2^)

**Table:**

	*x*	*y*	*z*	*U*_iso_*/*U*_eq_	
O1	0.81524 (10)	1.05479 (9)	0.57180 (7)	0.0297 (2)	
O2	0.75693 (9)	0.74987 (9)	−0.10597 (7)	0.0250 (2)	
O3	0.72502 (8)	0.57344 (8)	−0.08306 (7)	0.01937 (18)	
O4	0.51599 (9)	0.71161 (10)	0.19678 (7)	0.0273 (2)	
O5	0.52850 (8)	0.70160 (8)	0.03110 (7)	0.01941 (18)	
C1	0.77006 (10)	0.78011 (10)	0.32029 (9)	0.0143 (2)	
H1	0.6900	0.7604	0.3444	0.017*	
C2	0.83008 (11)	0.65818 (11)	0.26061 (9)	0.0155 (2)	
C3	0.77594 (11)	0.54883 (11)	0.25838 (10)	0.0189 (2)	
H3	0.7002	0.5521	0.2912	0.023*	
C4	0.82952 (12)	0.43446 (12)	0.20946 (11)	0.0239 (3)	
H4	0.7917	0.3611	0.2102	0.029*	
C5	0.93852 (13)	0.42927 (12)	0.15987 (11)	0.0268 (3)	
H5	0.9751	0.3515	0.1253	0.032*	
C6	0.99528 (12)	0.53692 (12)	0.16008 (11)	0.0229 (3)	
H6	1.0698	0.5324	0.1252	0.028*	
C7	0.94239 (11)	0.65107 (11)	0.21156 (9)	0.0174 (2)	
O8	0.99408 (8)	0.76150 (8)	0.21894 (7)	0.01886 (18)	
C9	1.10551 (11)	0.76050 (12)	0.16679 (11)	0.0224 (3)	
H9A	1.0964	0.7037	0.0902	0.027*	
H9B	1.1721	0.7320	0.1995	0.027*	
C10	1.13320 (11)	0.89193 (12)	0.18009 (10)	0.0221 (3)	
H10A	1.1332	0.9495	0.2565	0.027*	
H10B	1.2147	0.8986	0.1532	0.027*	
O11	1.04269 (8)	0.92460 (8)	0.12188 (7)	0.01924 (18)	
C12	1.02925 (12)	1.05545 (12)	0.16091 (11)	0.0231 (3)	
H12A	1.0920	1.0931	0.1312	0.028*	
H12B	1.0400	1.0943	0.2396	0.028*	
C13	0.90450 (12)	1.07848 (12)	0.12842 (11)	0.0232 (3)	
H13A	0.8981	1.1676	0.1429	0.028*	
H13B	0.8875	1.0279	0.0514	0.028*	
O14	0.82056 (8)	1.04353 (8)	0.19004 (7)	0.01873 (18)	
C15	0.69884 (11)	1.05639 (11)	0.17862 (9)	0.0171 (2)	
C16	0.64916 (13)	1.10929 (11)	0.11422 (10)	0.0219 (3)	
H16	0.7004	1.1358	0.0721	0.026*	
C17	0.52405 (13)	1.12324 (12)	0.11168 (11)	0.0251 (3)	
H17	0.4903	1.1604	0.0683	0.030*	
C18	0.44833 (12)	1.08351 (12)	0.17164 (11)	0.0237 (3)	
H18	0.3631	1.0939	0.1702	0.028*	
C19	0.49853 (11)	1.02807 (11)	0.23404 (10)	0.0194 (2)	
H19	0.4464	0.9996	0.2743	0.023*	
C20	0.62324 (11)	1.01343 (10)	0.23881 (9)	0.0155 (2)	
C21	0.67458 (10)	0.95795 (10)	0.31156 (9)	0.0142 (2)	
H21	0.6051	0.9316	0.3445	0.017*	
C22	0.75899 (11)	1.05411 (10)	0.40273 (9)	0.0148 (2)	
H22	0.8318	1.0718	0.3680	0.018*	
C23	0.80526 (11)	0.99655 (11)	0.47590 (9)	0.0173 (2)	
C24	0.84906 (10)	0.86418 (11)	0.42172 (9)	0.0152 (2)	
H24	0.9333	0.8709	0.3979	0.018*	
N25	0.74589 (9)	0.84833 (9)	0.25395 (7)	0.01416 (19)	
C26	0.69852 (10)	1.17631 (10)	0.45793 (9)	0.0154 (2)	
C27	0.72415 (11)	1.27232 (11)	0.42612 (9)	0.0181 (2)	
H27	0.7821	1.2609	0.3730	0.022*	
C28	0.66661 (12)	1.38434 (12)	0.47051 (10)	0.0222 (3)	
H28	0.6854	1.4489	0.4480	0.027*	
C29	0.58174 (12)	1.40171 (12)	0.54767 (10)	0.0228 (3)	
H29	0.5423	1.4783	0.5786	0.027*	
C30	0.55464 (11)	1.30695 (12)	0.57959 (10)	0.0217 (3)	
H30	0.4960	1.3186	0.6322	0.026*	
C31	0.61253 (11)	1.19494 (12)	0.53530 (9)	0.0191 (2)	
H31	0.5933	1.1306	0.5579	0.023*	
C32	0.85723 (11)	0.79886 (11)	0.49579 (9)	0.0162 (2)	
C33	0.75722 (12)	0.79095 (12)	0.55415 (10)	0.0202 (2)	
H33	0.6861	0.8338	0.5526	0.024*	
C34	0.76049 (13)	0.72117 (13)	0.61443 (10)	0.0257 (3)	
H34	0.6922	0.7170	0.6541	0.031*	
C35	0.86378 (14)	0.65763 (13)	0.61657 (11)	0.0276 (3)	
H35	0.8661	0.6094	0.6573	0.033*	
C36	0.96326 (13)	0.66473 (12)	0.55933 (11)	0.0255 (3)	
H36	1.0339	0.6211	0.5607	0.031*	
C37	0.96060 (11)	0.73542 (11)	0.49962 (10)	0.0196 (2)	
H37	1.0298	0.7405	0.4612	0.023*	
C38	0.70379 (10)	0.77556 (10)	0.14631 (9)	0.0145 (2)	
C39	0.77988 (11)	0.75955 (10)	0.06806 (9)	0.0156 (2)	
H39	0.8610	0.7924	0.0891	0.019*	
C40	0.75136 (10)	0.69577 (11)	−0.04851 (9)	0.0163 (2)	
C41	0.68623 (13)	0.51164 (13)	−0.19471 (10)	0.0246 (3)	
H41A	0.6577	0.4258	−0.2111	0.037*	
H41B	0.7549	0.5110	−0.2391	0.037*	
H41C	0.6198	0.5565	−0.2093	0.037*	
C42	0.57397 (11)	0.72587 (11)	0.12884 (9)	0.0166 (2)	
C43	0.40551 (12)	0.64856 (14)	0.00728 (11)	0.0258 (3)	
H43A	0.3821	0.6318	−0.0666	0.039*	
H43B	0.3501	0.7073	0.0556	0.039*	
H43C	0.4008	0.5705	0.0167	0.039*	

### Atomic displacement parameters (Å^2^)

**Table:**

	*U*^11^	*U*^22^	*U*^33^	*U*^12^	*U*^13^	*U*^23^
O1	0.0505 (6)	0.0199 (5)	0.0154 (4)	0.0054 (4)	−0.0083 (4)	0.0039 (4)
O2	0.0340 (5)	0.0243 (5)	0.0195 (4)	−0.0045 (4)	−0.0008 (4)	0.0124 (4)
O3	0.0254 (4)	0.0162 (4)	0.0143 (4)	−0.0019 (3)	−0.0010 (3)	0.0044 (3)
O4	0.0246 (5)	0.0382 (6)	0.0198 (4)	−0.0119 (4)	−0.0015 (4)	0.0140 (4)
O5	0.0159 (4)	0.0261 (5)	0.0174 (4)	−0.0025 (3)	−0.0027 (3)	0.0104 (4)
C1	0.0160 (5)	0.0134 (5)	0.0144 (5)	−0.0004 (4)	−0.0005 (4)	0.0069 (4)
C2	0.0175 (5)	0.0148 (5)	0.0139 (5)	0.0007 (4)	−0.0022 (4)	0.0058 (4)
C3	0.0205 (6)	0.0168 (5)	0.0194 (6)	−0.0011 (4)	−0.0018 (4)	0.0076 (5)
C4	0.0284 (7)	0.0145 (6)	0.0289 (7)	−0.0006 (5)	−0.0023 (5)	0.0092 (5)
C5	0.0314 (7)	0.0162 (6)	0.0309 (7)	0.0065 (5)	0.0028 (6)	0.0075 (5)
C6	0.0223 (6)	0.0214 (6)	0.0254 (6)	0.0051 (5)	0.0040 (5)	0.0095 (5)
C7	0.0193 (6)	0.0156 (5)	0.0181 (5)	0.0001 (4)	−0.0012 (4)	0.0078 (4)
O8	0.0176 (4)	0.0174 (4)	0.0226 (4)	0.0005 (3)	0.0043 (3)	0.0091 (3)
C9	0.0177 (6)	0.0265 (6)	0.0262 (6)	0.0031 (5)	0.0054 (5)	0.0137 (5)
C10	0.0178 (6)	0.0275 (7)	0.0227 (6)	−0.0040 (5)	−0.0008 (5)	0.0125 (5)
O11	0.0222 (4)	0.0162 (4)	0.0182 (4)	−0.0015 (3)	−0.0001 (3)	0.0062 (3)
C12	0.0260 (6)	0.0158 (6)	0.0256 (6)	−0.0035 (5)	0.0063 (5)	0.0065 (5)
C13	0.0295 (7)	0.0194 (6)	0.0257 (6)	0.0016 (5)	0.0093 (5)	0.0139 (5)
O14	0.0206 (4)	0.0205 (4)	0.0181 (4)	−0.0010 (3)	0.0021 (3)	0.0110 (3)
C15	0.0226 (6)	0.0121 (5)	0.0148 (5)	0.0008 (4)	−0.0011 (4)	0.0039 (4)
C16	0.0322 (7)	0.0166 (6)	0.0181 (6)	0.0000 (5)	−0.0013 (5)	0.0087 (5)
C17	0.0350 (7)	0.0190 (6)	0.0226 (6)	0.0040 (5)	−0.0058 (5)	0.0099 (5)
C18	0.0240 (6)	0.0207 (6)	0.0251 (6)	0.0043 (5)	−0.0051 (5)	0.0082 (5)
C19	0.0221 (6)	0.0162 (5)	0.0183 (6)	0.0013 (4)	−0.0009 (5)	0.0055 (5)
C20	0.0213 (6)	0.0112 (5)	0.0128 (5)	0.0008 (4)	−0.0022 (4)	0.0039 (4)
C21	0.0161 (5)	0.0138 (5)	0.0127 (5)	0.0000 (4)	−0.0004 (4)	0.0056 (4)
C22	0.0174 (5)	0.0136 (5)	0.0131 (5)	−0.0007 (4)	−0.0006 (4)	0.0054 (4)
C23	0.0198 (6)	0.0155 (5)	0.0163 (5)	−0.0018 (4)	−0.0029 (4)	0.0066 (4)
C24	0.0163 (5)	0.0156 (5)	0.0144 (5)	−0.0002 (4)	−0.0009 (4)	0.0071 (4)
N25	0.0176 (5)	0.0122 (4)	0.0120 (4)	0.0008 (3)	−0.0016 (3)	0.0044 (4)
C26	0.0172 (5)	0.0135 (5)	0.0133 (5)	−0.0008 (4)	−0.0025 (4)	0.0037 (4)
C27	0.0210 (6)	0.0168 (5)	0.0161 (5)	−0.0003 (4)	0.0020 (4)	0.0064 (4)
C28	0.0293 (7)	0.0168 (6)	0.0208 (6)	0.0009 (5)	−0.0016 (5)	0.0081 (5)
C29	0.0256 (6)	0.0197 (6)	0.0189 (6)	0.0067 (5)	−0.0014 (5)	0.0035 (5)
C30	0.0182 (6)	0.0260 (6)	0.0168 (6)	0.0011 (5)	0.0010 (4)	0.0047 (5)
C31	0.0217 (6)	0.0195 (6)	0.0159 (5)	−0.0023 (4)	0.0001 (4)	0.0074 (5)
C32	0.0203 (6)	0.0140 (5)	0.0139 (5)	−0.0014 (4)	−0.0030 (4)	0.0055 (4)
C33	0.0219 (6)	0.0195 (6)	0.0179 (6)	0.0000 (5)	0.0005 (5)	0.0064 (5)
C34	0.0335 (7)	0.0241 (6)	0.0193 (6)	−0.0071 (5)	0.0014 (5)	0.0093 (5)
C35	0.0405 (8)	0.0204 (6)	0.0255 (7)	−0.0078 (5)	−0.0087 (6)	0.0143 (5)
C36	0.0290 (7)	0.0181 (6)	0.0294 (7)	−0.0010 (5)	−0.0103 (5)	0.0103 (5)
C37	0.0197 (6)	0.0172 (5)	0.0204 (6)	−0.0003 (4)	−0.0031 (5)	0.0065 (5)
C38	0.0173 (5)	0.0121 (5)	0.0143 (5)	−0.0005 (4)	−0.0020 (4)	0.0060 (4)
C39	0.0170 (5)	0.0135 (5)	0.0164 (5)	−0.0003 (4)	−0.0014 (4)	0.0067 (4)
C40	0.0140 (5)	0.0180 (5)	0.0163 (5)	0.0001 (4)	0.0020 (4)	0.0064 (4)
C41	0.0307 (7)	0.0230 (6)	0.0148 (6)	−0.0048 (5)	−0.0036 (5)	0.0030 (5)
C42	0.0189 (5)	0.0140 (5)	0.0166 (5)	−0.0003 (4)	−0.0009 (4)	0.0061 (4)
C43	0.0185 (6)	0.0328 (7)	0.0259 (7)	−0.0056 (5)	−0.0059 (5)	0.0127 (6)

### Geometric parameters (Å, º)

**Table:**

O1—C23	1.2125 (15)	C19—C20	1.3909 (17)
O2—C40	1.2048 (14)	C19—H19	0.9500
O3—C40	1.3378 (14)	C20—C21	1.5135 (15)
O3—C41	1.4511 (14)	C21—N25	1.4717 (14)
O4—C42	1.2072 (15)	C21—C22	1.5584 (16)
O5—C42	1.3373 (14)	C21—H21	1.0000
O5—C43	1.4422 (15)	C22—C26	1.5101 (16)
C1—N25	1.4703 (14)	C22—C23	1.5164 (16)
C1—C2	1.5141 (16)	C22—H22	1.0000
C1—C24	1.5610 (16)	C23—C24	1.5269 (16)
C1—H1	1.0000	C24—C32	1.5129 (15)
C2—C3	1.3892 (16)	C24—H24	1.0000
C2—C7	1.4081 (17)	N25—C38	1.4297 (14)
C3—C4	1.3935 (17)	C26—C31	1.3934 (16)
C3—H3	0.9500	C26—C27	1.3939 (16)
C4—C5	1.382 (2)	C27—C28	1.3881 (17)
C4—H4	0.9500	C27—H27	0.9500
C5—C6	1.3934 (19)	C28—C29	1.3847 (19)
C5—H5	0.9500	C28—H28	0.9500
C6—C7	1.3918 (17)	C29—C30	1.3844 (19)
C6—H6	0.9500	C29—H29	0.9500
C7—O8	1.3656 (14)	C30—C31	1.3898 (18)
O8—C9	1.4308 (14)	C30—H30	0.9500
C9—C10	1.5025 (18)	C31—H31	0.9500
C9—H9A	0.9900	C32—C37	1.3935 (17)
C9—H9B	0.9900	C32—C33	1.3982 (17)
C10—O11	1.4240 (15)	C33—C34	1.3892 (18)
C10—H10A	0.9900	C33—H33	0.9500
C10—H10B	0.9900	C34—C35	1.388 (2)
O11—C12	1.4234 (15)	C34—H34	0.9500
C12—C13	1.5013 (19)	C35—C36	1.381 (2)
C12—H12A	0.9900	C35—H35	0.9500
C12—H12B	0.9900	C36—C37	1.3914 (18)
C13—O14	1.4268 (14)	C36—H36	0.9500
C13—H13A	0.9900	C37—H37	0.9500
C13—H13B	0.9900	C38—C39	1.3335 (16)
O14—C15	1.3647 (15)	C38—C42	1.4995 (16)
C15—C16	1.3901 (16)	C39—C40	1.4866 (16)
C15—C20	1.4070 (16)	C39—H39	0.9500
C16—C17	1.3921 (19)	C41—H41A	0.9800
C16—H16	0.9500	C41—H41B	0.9800
C17—C18	1.384 (2)	C41—H41C	0.9800
C17—H17	0.9500	C43—H43A	0.9800
C18—C19	1.3919 (17)	C43—H43B	0.9800
C18—H18	0.9500	C43—H43C	0.9800
			
C40—O3—C41	114.59 (9)	C26—C22—C21	111.27 (9)
C42—O5—C43	115.98 (10)	C23—C22—C21	110.24 (9)
N25—C1—C2	113.05 (9)	C26—C22—H22	106.6
N25—C1—C24	110.14 (9)	C23—C22—H22	106.6
C2—C1—C24	110.35 (9)	C21—C22—H22	106.6
N25—C1—H1	107.7	O1—C23—C22	122.18 (11)
C2—C1—H1	107.7	O1—C23—C24	121.31 (11)
C24—C1—H1	107.7	C22—C23—C24	116.32 (10)
C3—C2—C7	118.04 (11)	C32—C24—C23	113.21 (9)
C3—C2—C1	119.03 (10)	C32—C24—C1	108.91 (9)
C7—C2—C1	122.84 (10)	C23—C24—C1	113.21 (9)
C2—C3—C4	121.92 (12)	C32—C24—H24	107.0
C2—C3—H3	119.0	C23—C24—H24	107.0
C4—C3—H3	119.0	C1—C24—H24	107.0
C5—C4—C3	119.04 (12)	C38—N25—C1	116.67 (9)
C5—C4—H4	120.5	C38—N25—C21	114.50 (9)
C3—C4—H4	120.5	C1—N25—C21	110.81 (9)
C4—C5—C6	120.66 (12)	C31—C26—C27	118.32 (11)
C4—C5—H5	119.7	C31—C26—C22	122.69 (10)
C6—C5—H5	119.7	C27—C26—C22	118.88 (10)
C7—C6—C5	119.73 (12)	C28—C27—C26	121.26 (11)
C7—C6—H6	120.1	C28—C27—H27	119.4
C5—C6—H6	120.1	C26—C27—H27	119.4
O8—C7—C6	123.51 (11)	C29—C28—C27	119.77 (12)
O8—C7—C2	115.92 (10)	C29—C28—H28	120.1
C6—C7—C2	120.57 (11)	C27—C28—H28	120.1
C7—O8—C9	118.88 (9)	C30—C29—C28	119.69 (12)
O8—C9—C10	106.65 (10)	C30—C29—H29	120.2
O8—C9—H9A	110.4	C28—C29—H29	120.2
C10—C9—H9A	110.4	C29—C30—C31	120.50 (12)
O8—C9—H9B	110.4	C29—C30—H30	119.7
C10—C9—H9B	110.4	C31—C30—H30	119.7
H9A—C9—H9B	108.6	C30—C31—C26	120.46 (11)
O11—C10—C9	108.74 (10)	C30—C31—H31	119.8
O11—C10—H10A	109.9	C26—C31—H31	119.8
C9—C10—H10A	109.9	C37—C32—C33	118.52 (11)
O11—C10—H10B	109.9	C37—C32—C24	120.51 (11)
C9—C10—H10B	109.9	C33—C32—C24	120.70 (11)
H10A—C10—H10B	108.3	C34—C33—C32	120.85 (12)
C12—O11—C10	113.17 (10)	C34—C33—H33	119.6
O11—C12—C13	108.94 (10)	C32—C33—H33	119.6
O11—C12—H12A	109.9	C35—C34—C33	119.87 (13)
C13—C12—H12A	109.9	C35—C34—H34	120.1
O11—C12—H12B	109.9	C33—C34—H34	120.1
C13—C12—H12B	109.9	C36—C35—C34	119.87 (12)
H12A—C12—H12B	108.3	C36—C35—H35	120.1
O14—C13—C12	106.48 (10)	C34—C35—H35	120.1
O14—C13—H13A	110.4	C35—C36—C37	120.37 (12)
C12—C13—H13A	110.4	C35—C36—H36	119.8
O14—C13—H13B	110.4	C37—C36—H36	119.8
C12—C13—H13B	110.4	C36—C37—C32	120.52 (12)
H13A—C13—H13B	108.6	C36—C37—H37	119.7
C15—O14—C13	119.73 (10)	C32—C37—H37	119.7
O14—C15—C16	124.16 (11)	C39—C38—N25	118.66 (10)
O14—C15—C20	115.34 (10)	C39—C38—C42	124.16 (10)
C16—C15—C20	120.50 (11)	N25—C38—C42	117.15 (10)
C15—C16—C17	119.74 (12)	C38—C39—C40	126.99 (11)
C15—C16—H16	120.1	C38—C39—H39	116.5
C17—C16—H16	120.1	C40—C39—H39	116.5
C18—C17—C16	120.65 (12)	O2—C40—O3	123.95 (11)
C18—C17—H17	119.7	O2—C40—C39	122.18 (11)
C16—C17—H17	119.7	O3—C40—C39	113.80 (10)
C17—C18—C19	119.21 (12)	O3—C41—H41A	109.5
C17—C18—H18	120.4	O3—C41—H41B	109.5
C19—C18—H18	120.4	H41A—C41—H41B	109.5
C20—C19—C18	121.55 (12)	O3—C41—H41C	109.5
C20—C19—H19	119.2	H41A—C41—H41C	109.5
C18—C19—H19	119.2	H41B—C41—H41C	109.5
C19—C20—C15	118.32 (11)	O4—C42—O5	123.60 (11)
C19—C20—C21	119.78 (11)	O4—C42—C38	123.36 (11)
C15—C20—C21	121.84 (10)	O5—C42—C38	113.02 (10)
N25—C21—C20	112.36 (9)	O5—C43—H43A	109.5
N25—C21—C22	107.51 (9)	O5—C43—H43B	109.5
C20—C21—C22	111.79 (9)	H43A—C43—H43B	109.5
N25—C21—H21	108.4	O5—C43—H43C	109.5
C20—C21—H21	108.4	H43A—C43—H43C	109.5
C22—C21—H21	108.4	H43B—C43—H43C	109.5
C26—C22—C23	115.04 (9)		
			
N25—C1—C2—C3	−125.05 (11)	N25—C1—C24—C32	171.97 (9)
C24—C1—C2—C3	111.10 (12)	C2—C1—C24—C32	−62.52 (12)
N25—C1—C2—C7	58.41 (14)	N25—C1—C24—C23	45.09 (13)
C24—C1—C2—C7	−65.44 (14)	C2—C1—C24—C23	170.60 (9)
C7—C2—C3—C4	−0.05 (18)	C2—C1—N25—C38	39.74 (13)
C1—C2—C3—C4	−176.75 (11)	C24—C1—N25—C38	163.70 (9)
C2—C3—C4—C5	−1.30 (19)	C2—C1—N25—C21	173.16 (9)
C3—C4—C5—C6	1.0 (2)	C24—C1—N25—C21	−62.87 (12)
C4—C5—C6—C7	0.6 (2)	C20—C21—N25—C38	−32.35 (13)
C5—C6—C7—O8	177.52 (12)	C22—C21—N25—C38	−155.76 (9)
C5—C6—C7—C2	−2.00 (19)	C20—C21—N25—C1	−166.85 (9)
C3—C2—C7—O8	−177.85 (10)	C22—C21—N25—C1	69.74 (11)
C1—C2—C7—O8	−1.28 (16)	C23—C22—C26—C31	−46.14 (15)
C3—C2—C7—C6	1.70 (17)	C21—C22—C26—C31	80.13 (13)
C1—C2—C7—C6	178.27 (11)	C23—C22—C26—C27	137.69 (11)
C6—C7—O8—C9	3.25 (17)	C21—C22—C26—C27	−96.03 (12)
C2—C7—O8—C9	−177.21 (10)	C31—C26—C27—C28	0.48 (18)
C7—O8—C9—C10	174.76 (10)	C22—C26—C27—C28	176.81 (11)
O8—C9—C10—O11	−66.55 (13)	C26—C27—C28—C29	−0.20 (19)
C9—C10—O11—C12	156.09 (10)	C27—C28—C29—C30	−0.26 (19)
C10—O11—C12—C13	−156.89 (10)	C28—C29—C30—C31	0.43 (19)
O11—C12—C13—O14	70.43 (12)	C29—C30—C31—C26	−0.14 (19)
C12—C13—O14—C15	178.94 (10)	C27—C26—C31—C30	−0.31 (18)
C13—O14—C15—C16	−4.04 (17)	C22—C26—C31—C30	−176.49 (11)
C13—O14—C15—C20	176.88 (10)	C23—C24—C32—C37	−132.76 (11)
O14—C15—C16—C17	−177.13 (11)	C1—C24—C32—C37	100.36 (12)
C20—C15—C16—C17	1.91 (18)	C23—C24—C32—C33	53.27 (15)
C15—C16—C17—C18	−0.82 (19)	C1—C24—C32—C33	−73.60 (13)
C16—C17—C18—C19	−0.6 (2)	C37—C32—C33—C34	−0.18 (18)
C17—C18—C19—C20	1.01 (19)	C24—C32—C33—C34	173.90 (11)
C18—C19—C20—C15	0.06 (17)	C32—C33—C34—C35	−0.40 (19)
C18—C19—C20—C21	177.24 (11)	C33—C34—C35—C36	0.4 (2)
O14—C15—C20—C19	177.59 (10)	C34—C35—C36—C37	0.1 (2)
C16—C15—C20—C19	−1.53 (17)	C35—C36—C37—C32	−0.70 (19)
O14—C15—C20—C21	0.48 (16)	C33—C32—C37—C36	0.73 (18)
C16—C15—C20—C21	−178.64 (11)	C24—C32—C37—C36	−173.36 (11)
C19—C20—C21—N25	124.26 (11)	C1—N25—C38—C39	−107.40 (12)
C15—C20—C21—N25	−58.67 (14)	C21—N25—C38—C39	120.86 (11)
C19—C20—C21—C22	−114.76 (12)	C1—N25—C38—C42	74.83 (13)
C15—C20—C21—C22	62.31 (14)	C21—N25—C38—C42	−56.91 (13)
N25—C21—C22—C26	173.02 (9)	N25—C38—C39—C40	−175.09 (10)
C20—C21—C22—C26	49.26 (13)	C42—C38—C39—C40	2.52 (19)
N25—C21—C22—C23	−58.10 (11)	C41—O3—C40—O2	−8.29 (17)
C20—C21—C22—C23	178.15 (9)	C41—O3—C40—C39	174.69 (10)
C26—C22—C23—O1	−14.07 (17)	C38—C39—C40—O2	116.68 (14)
C21—C22—C23—O1	−140.87 (12)	C38—C39—C40—O3	−66.24 (15)
C26—C22—C23—C24	170.95 (10)	C43—O5—C42—O4	−3.85 (17)
C21—C22—C23—C24	44.14 (13)	C43—O5—C42—C38	177.49 (10)
O1—C23—C24—C32	22.51 (16)	C39—C38—C42—O4	159.93 (12)
C22—C23—C24—C32	−162.46 (10)	N25—C38—C42—O4	−22.44 (17)
O1—C23—C24—C1	147.08 (12)	C39—C38—C42—O5	−21.41 (16)
C22—C23—C24—C1	−37.89 (14)	N25—C38—C42—O5	156.22 (10)

### Hydrogen-bond geometry (Å, º)

**Table:**

*D*—H···*A*	*D*—H	H···*A*	*D*···*A*	*D*—H···*A*
C6—H6···O3^i^	0.95	2.58	3.3982 (16)	145
C10—H10*A*···O1^ii^	0.99	2.44	3.2433 (16)	138
C12—H12*A*···O2^iii^	0.99	2.58	3.5345 (18)	162
C17—H17···O5^iv^	0.95	2.53	3.4409 (18)	160
C30—H30···O4^v^	0.95	2.53	3.2834 (16)	136
C41—H41*A*···O4^vi^	0.98	2.52	3.3758 (19)	145

Symmetry codes: (i) −*x*+2, −*y*+1, −*z*; (ii) −*x*+2, −*y*+2, −*z*+1; (iii) −*x*+2, −*y*+2, −*z*; (iv) −*x*+1, −*y*+2, −*z*; (v) −*x*+1, −*y*+2, −*z*+1; (vi) −*x*+1, −*y*+1, −*z*.
